# Supplementary material for: Next-generation sequencing-based gene panel tests for the detection of rare variants and hypomorphic alleles associated with primary open-angle glaucoma
Source: PLoS One. 2024 Jan 19;19(1):e0282133. doi: 10.1371/journal.pone.0282133 (PMC10798505; doi:10.1371/journal.pone.0282133)
Supplement: S1 Table — (DOCX) [file pone.0282133.s001.docx]

**S1 Table**. Primers designed to amplify coding region of the *CYP1B1* gene.

| **Codificant exons of *CYP1B1*** | **Forward primer** | **Reverse primer** | **Product size** |
| --- | --- | --- | --- |
| **Exon 2** | 5’ CTGTCTCTGCACCCCTGAG 3’ | 5’ CGAAACACACGGCACTCAT 3’ | 679 bp |
|  | 5’ CTACTCGGAGCACTGGAAGG 3’ | 5’ TCTCTACTCCGCCTTTTTCA 3’ | 696 bp |
| **Exon 3** | 5’ GAATTTTGCTCACTTGCTTTTC 3’ | 5’ AGCTCCTGCATAGCCCACTA 3’ | 830 bp |
